# Management of Rat Lungworm Disease (Neuroangiostrongyliasis) Using Anthelmintics: Recent Updates and Recommendations

**DOI:** 10.3390/pathogens12010023

**Published:** 2022-12-23

**Authors:** John Jacob, Argon Steel, Kathleen Howe, Susan Jarvi

**Affiliations:** Department of Pharmaceutical Sciences, Daniel K. Inouye College of Pharmacy, University of Hawaii at Hilo, Hilo, HI 96720, USA

**Keywords:** rat lungworm, neuroangiostrongyliasis, treatment, anthelmintics, albendazole, pyrantel pamoate

## Abstract

While there have been legitimate concerns in the past regarding the use of anthelmintics for the management of neuroangiostrongyliasis (rat lungworm disease), recent studies demonstrate that they can be considered safe and efficacious, particularly albendazole, which is regarded as the choice anthelmintic for its management. However, physician hesitancy to prescribe, as well as problems of availability persist, at least in Hawaii, which is considered the epicenter of this disease in the US. As a result, many patients suffer a diminished quality of life or even death. Here, we discuss recent studies that provide insights into new treatments and preventative interventions, which can be more rigorously used for the management of neuroangiostrongyliasis. In summary, results from recent studies suggest that albendazole and avermectins are beneficial for post-exposure management, pyrantel pamoate is beneficial as a post-exposure prophylactic, and levamisole is deserving of further study for the treatment of neuroangiostrongyliasis.

## 1. Introduction

The use of anthelmintics for the management of neuroangiostrongyliasis (rat lungworm disease) has been historically controversial due to the theoretical concern that killing *Angiostrongyliasis cantonensis* (rat lungworm) larvae that have migrated into the central nervous system (CNS) could trigger a severe inflammatory response, resulting in exacerbation of symptoms and further complications [1,2,3]. Such concerns have made many clinicians hesitant to consider such therapeutic interventions, despite the lack of actual evidence to validate this theory.

A further argument against the use of anthelmintics questions their necessity. Historically, the first human neuroangiostrongyliasis cases were reported from Asian countries and were associated with headaches and other mild symptoms, were typically self-resolving [4,5,6], or were successfully treated with only corticosteroids [4,7,8]. This led many clinicians in the West, particularly in the US, to expect a similar prognosis. In fact, this disease has been historically considered flu-like and self-resolving [4,5,6,9], and has given rise to the misperceptions among US clinicians that anthelminthic drugs were unnecessary or to wait until all other treatment options have been exhausted [10,11,12].

We believe that these arguments against the use of anthelmintics should be further examined. Primarily, it has become clear that cases in the US are often quite severe—to the extent of being fatal [10,11,12,13]. While the reasons for a geographical variation in disease severity remain unclear, the expectation that neuroangiostrongyliasis cases will self-resolve is no longer a tenable reason for delaying treatment. Secondly, it is worth noting that numerous other cerebral parasitic infections that can also cause eosinophilic meningitis are routinely treated with anthelmintics. For instance, a high dose of albendazole is used for the management of baylisascariasis, toxocariasis, gnathostomiasis, and cysticercosis (cestode), and similarly, a high dose of praziquantel is used for the management of paragonimiasis and schistosomiasis [14].

Finally, our understanding of anthelmintics used for neuroangiostrongyliasis has improved in recent years. Several in vitro, in vivo, and clinical studies have confirmed the efficacy of albendazole and pyrantel pamoate as treatments for neuroangiostrongyliasis, with avermectins and levamisole emerging as promising candidates [15,16,17]. These insights have provided us with a much-improved understanding of these drugs’ safety and efficacy, allowing us to correct misconceptions and better manage this disease. Although the drugs mentioned above are classified as anthelmintics, each has unique therapeutic features and limitations, which we review below and summarize in Table 1.

### 1.1. Albendazole

Albendazole is a benzimidazole anthelmintic and, to date, is the most suitable anthelmintic for the management of neuroangiostrongyliasis due to its broad spectrum of nematocidal activity and ability to cross the blood–brain barrier (BBB) [18]. A systematic literature survey by Jacob et al. (2022a) [19] on the clinical outcomes associated with benzimidazole treatment found no evidence of albendazole resulting in cerebral inflammation or exacerbation of symptoms among patients with confirmed diagnoses of neuroangiostrongyliasis. This survey included an estimated 1034 patients and 2561 animals and provides highly supportive evidence for the safe and effective use of albendazole–corticosteroid co-therapy. The estimated dose of albendazole reported in these studies was approximately 15 mg/kg/day or 400 mg twice daily (with an average body weight of 60 kg) [19]. While bone marrow suppression and associated symptoms have been reported with long-term use of albendazole, overall, albendazole is generally considered to be a very safe anthelmintic [20]. Furthermore, no such side effects have been reported among neuroangiostrongyliasis patients [19].

Recent changes in clinical guidelines are also shifting in favor of using albendazole in the treatment of neuroangiostrongyliasis. Two hospitals in Australia (Sydney Children’s Hospital and Children’s Health Queensland Hospital) recommend using albendazole for the early management of neuroangiostrongyliasis in pediatrics [21,22]. Similarly, in the USA, Hilo Medical Center Hospital and the Hawaii Governor’s Rat Lungworm Taskforce also endorse the use of albendazole for the management of neuroangiostrongyliasis in adults [23,24].

#### Other Issues with Albendazole

In addition to the therapeutic concerns discussed above, other pharmacoeconomic and availability issues continue to hinder the broader adoption of albendazole in treating neuroangiostrongyliasis. For example, albendazole is one of the most expensive drugs on the US market, with a price estimated between USD 200-250/unit dose [25]. Since the use of albendazole for neuroangiostrongyliasis is still controversial and has not been approved by the US FDA, most insurance companies will not cover the cost of albendazole. According to the guidelines mentioned above [23,24], treatment for neuroangiostrongyliasis requires albendazole to be administered two times a day (BID) for 2–3 weeks, which means that the patient will have to personally pay an amount between USD 6000-9000 (i.e., 24–36-unit doses) just to cover the cost of albendazole.

Additionally, even in the scenario where the clinician is willing to prescribe albendazole and the patient’s insurance company is willing to cover its cost, due to its high price and relatively infrequent demand, most pharmacies do not stock sufficient quantities for adequate treatment. Many patients have experienced delayed access to albendazole due to the above reasons, resulting in life-long neurological sequelae and an associated decline in their quality of life (personal communications). Out of desperation, some of these patients have tried to self-medicate using veterinary formulations of albendazole, which are readily available and cost only a small fraction of the human formulations (USD 20-50). Given problems with self-diagnosis and calculating proper dosage, the use of veterinary anthelmintics in humans is highly problematic. Alternatively, some patients acquire personal stocks of albendazole by ordering it from countries such as India and Thailand, where the price of this drug is considerably lower.

We suggest that this availability issue could be resolved by having corporate pharmacies establish centralized stocking systems for albendazole in endemic areas, thus ensuring that sufficient quantities are routinely available.

### 1.2. Pyrantel Pamoate

Pyrantel pamoate’s potential efficacy against *A. cantonensis* has warranted discussion in previous studies [15,17,26], and the in vivo efficacy of this drug has recently been evaluated in an experimental rat model [27]. The findings suggest pyrantel pamoate to be an effective post-exposure prophylactic against neuroangiostrongyliasis by reducing the worm burden as well as delaying the establishment of infection, thus providing time for the administration of albendazole [27]. However, it should be emphasized that pyrantel pamoate is a luminal drug with activity limited to the gastrointestinal tract (GIT) and is only efficacious while the parasite is within the GIT. Once the parasite has entered systemic circulation, the drug is clinically ineffective [28,29]. Upon release of the results of the in vitro study in 2021 [17], Hilo Medical Center Hospital, Hilo, Hawaii, USA, immediately adopted the use of pyrantel pamoate as a post-exposure prophylactic in their clinical treatment guidelines [24]. This guideline recommends administering pyrantel pamoate as instructed by the manufacturer (the same dosage as for pinworm management), which is typically 11 mg/kg, depending on the manufacturer [30].

Pyrantel pamoate is available over the counter (OTC) from most pharmacies with an estimated cost ranging between USD 10–20 per dose. Since the prophylactic activity of pyrantel pamoate against *A. cantonensis* is a recent discovery [27], clinical data are not yet available.

### 1.3. Ivermectin

According to the literature, ivermectin [31,32] and levamisole [33,34] are the most widely used anthelmintics after benzimidazoles for the management of neuroangiostrongyliasis, and both drugs appear efficacious. 

Ivermectin does not directly kill the nematode; its paralyzing effect delays the progression of the infection and, to some extent, eradicates the parasite via immune responses and hepatic clearance. However, avermectins do not cross the BBB [35], and therefore, once *A. cantonensis* has entered the CNS, the drug is expected to be clinically ineffective. Thus, ivermectin is only efficacious during the early stages of infection when the parasite is within the GIT or systemic circulation. In theory, introducing ivermectin to an albendazole–corticosteroid co-therapy might produce a synergistic effect by paralyzing the nematode, slowing the progression of infection, and simultaneously increasing the exposure time to the nematocidal effects of albendazole [17]. Such synergistic effects of multiple anthelmintics have proven efficacious against Bancroftian filariasis, another parasitic nematode [36]. Future studies should investigate and compare the efficacy of the ivermectin–albendazole–corticosteroid cocktail with albendazole–corticosteroid co-therapy.

### 1.4. Levamisole

Experimental animal studies have shown levamisole to significantly reduce worm/larval burden, with the earliest administration (1–5 days post-infection) showing the most efficacy [37,38,39,40,41]. However, due to side effects such as agranulocytosis and its use as a cocaine adulterant, levamisole has been withdrawn from many global markets and is no longer available in many countries, including the US [42,43]. As shown in Table 1, since levamisole appears beneficial for the management of neuroangiostrongyliasis in humans [5,33], further research seems worthwhile.

**Table 1:** Summary of possible anthelmintic interventions for the management of rat lungworm disease (neuroangiostrongyliasis).

Anthelmintic	Mechanism of Action	Advantages	Disadvantages	References	Recommending Guidelines
Albendazole	Antimitotic	Larva/wormicide: kills the parasite Crosses the BBB	Slow acting Very expensive (in the USA) May not be readily available for purchase Prescription required	Jacob et al., 2021 [17] Jacob et al., 2022a [19] Jacob et al., 2022b [44]	Pediatric: Children’s Health Queensland Hospital [21] Sydney Children’s Hospital [22] Adult: Hawaii Governor’s Taskforce [23] Hilo Medical Center Hospital [24]
Pyrantel pamoate	Nicotinic agonist	Prophylactic Rapid acting Easily affordable Prescription not required (over-the-counter)	Works for only a short duration post-exposure * Temporary paralysis of the parasite Ineffective once the parasites are in the systemic circulation Does not cross BBB	Jacob et al., 2021 [17] Jacob et al., 2022c [27]	Hilo Medical Center Hospital [24]
Ivermectin	GABA agonist	Rapid acting Comparatively affordable	May not be readily available for purchase Prescription required Temporary paralysis of the parasite Does not cross BBB Ineffective once the parasites are in the CNS	Jacob et al., 2021 [17] Monteiro et al., 2020 [31] Defo et al. *,* 2018 [32]	N/A
Levamisole	Nicotinic agonist	Rapid acting	Withdrawn from most global markets (Unavailable in the USA) Prescription required Temporary paralysis of the parasite Does not cross BBB Ineffective once the parasites are in the CNS	Jacob et al., 2021 [17] Hwang et al., 1994 [34] Ma et al., 2018 [33]	N/A

BBB: Blood–brain barrier; GABA: gamma-aminobutyric acid; CNS: Central nervous system; N/A: none available; * further research is required to estimate the time.

## 2. Conclusions

While there have been legitimate concerns in the past regarding the use of anthelmintics, recent studies demonstrate that they can be considered safe and efficacious for the management of neuroangiostrongyliasis. Additionally, these recent studies also provide insights into more effective management of neuroangiostrongyliasis. Furthermore, attention needs to be directed toward their pharmacoeconomic and availability aspects, which vary widely among these anthelmintics. In summary, results from past and current studies suggest that albendazole and avermectins are beneficial for post-exposure management, pyrantel pamoate is beneficial as a post-exposure prophylactic, and levamisole appears deserving of further research for the treatment of neuroangiostrongyliasis. 
